# Volatiles from the xylarialean fungus *Hypoxylon invadens*

**DOI:** 10.3762/bjoc.14.62

**Published:** 2018-03-29

**Authors:** Jeroen S Dickschat, Tao Wang, Marc Stadler

**Affiliations:** 1 Kekulé-Institut für Organische Chemie, Universität Bonn, Gerhard-Domagk-Straße 1, 53121 Bonn, Germany; 2 Abteilung Mikrobielle Wirkstoffe, Helmholtz-Zentrum für Infektionsforschung, Inhoffenstraße 7, 38124 Braunschweig, Germany

**Keywords:** constitutional isomerism, gas chromatography, mass spectrometry, natural products, volatiles

## Abstract

The volatiles emitted by agar plate cultures of the xylarialean fungus *Hypoxylon invadens* were investigated by use of a closed loop stripping apparatus in combination with GC–MS. Several aromatic compounds were found that could only be identified by comparison to all possible constitutional isomers with different ring substitution patterns. For the set of identified compounds a plausible biosynthetic scheme was suggested that gives further support for the assigned structures.

## Introduction

**Figure 1:**
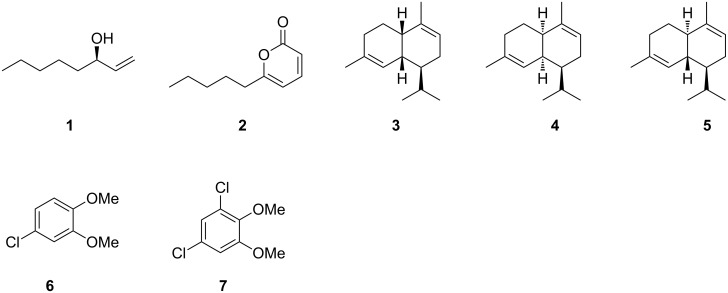
Structures of the widespread fungal volatiles oct-1-en-3-ol (**1**) and 6-pentyl-2*H*-pyran-2-one (**2**), the stereoisomers α-muurolene (**3**), α-amorphene (**4**) and α-cadinene (**5**), and the chlorinated aromatic compounds 1-chloro-3,4-dimethoxybenzene (**6**) and 1,3-dichloro-4,5-dimethoxybenzene (**7**) from *Geniculosporium*.

The research during the past decades has shown that many fungi release a rich bouquet of volatile organic compounds [1]. Some of these metabolites contribute to the pleasant aroma of edible mushrooms, e.g., the widespread compound oct-1-en-3-ol (**1**) is responsible for the typical odour of the button mushroom, *Agaricus bisporus*, and other delicacies from the fungal world such as the oyster mushroom, the penny bun, and shiitake [2–3]. The ecological function of most fungal volatiles is unknown, but for the alcohol **1** (Figure 1) a germination inhibitory function has been reported [4]. For 6-pentyl-2*H*-pyran-2-one (**2**), another widespread fungal volatile, a plant growth promoting effect and an induction of systemic resistance in plants against fungi was observed [5]. The significant biological effects of these and other fungal volatiles recently resulted in a considerable interest of natural product chemists and ecologists in volatile secondary metabolites.

Volatile natural products can efficiently be captured on charcoal filter traps by using a closed-loop stripping apparatus (CLSA) [6] or on polydimethylsiloxane fibres by application of the solid phase micro-extraction method (SPME) [7], followed by GC–MS analysis of the obtained extracts [8]. Compound identification is then usually performed by comparison of measured mass spectra to mass spectra in electronic libraries, in addition to comparison of measured to published retention indices. Positive compound identification can be assumed, if the mass spectrum and the retention index match reported data. The comparison of retention indices is particularly important, if different stereoisomers need to be considered, because stereoisomers may have very similar mass spectra, as observed for the sesquiterpenes α-muurolene (**3**), α-amorphene (**4**), and α-cadinene (**5**). The same problem applies to the unambiguous identification of regioisomers of aromatic compounds. We have recently reported on the GC–MS-based identification of the fungal volatiles 1-chloro-3,4-dimethoxybenzene (**6**) and 1,3-dichloro-4,5-dimethoxybenzene (**7**) from an endophytic *Geniculosporium* sp. by comparison of the natural products to all possible regioisomers that were obtained by chemical synthesis [9]. Here we report on the identification of the volatiles emitted by the xylarialean fungus *Hypoxylon invadens* MUCL 54175, a highly interesting pyrenomycete that was recently described as a new species [10]. This species is apparently rare and has hitherto only been found twice in Southwestern France, colonising the stromata (fruiting bodies) of another species of the same genus, the ubiquitous *Hypoxylon fragiforme*. The genus *Hypoxylon* was traditionally accommodated in the family Xylariaceae, but has recently been reassigned to the Hypoxylaceae. This family was resurrected as a result of intensive polyphasic studies on the biological and chemical diversity of the ascomycete order Xylariales, which is well-known for its diversity of bioactive secondary metabolites [11–12].

We decided to select a culture initiated from the germinating ascospores of *H. invadens* among a panel of hypoxylaceous fungi that were studied for comparison of their volatile profiles [13]. Several aromatic compounds were detected in the headspace extracts of *Hypoxylon invadens* MUCL 54175 for which an unambiguous GC–MS-based structural assignment was only possible by comparison to all regioisomers with different substitution patterns at the benzene ring.

## Results and Discussion

**Figure 2:**
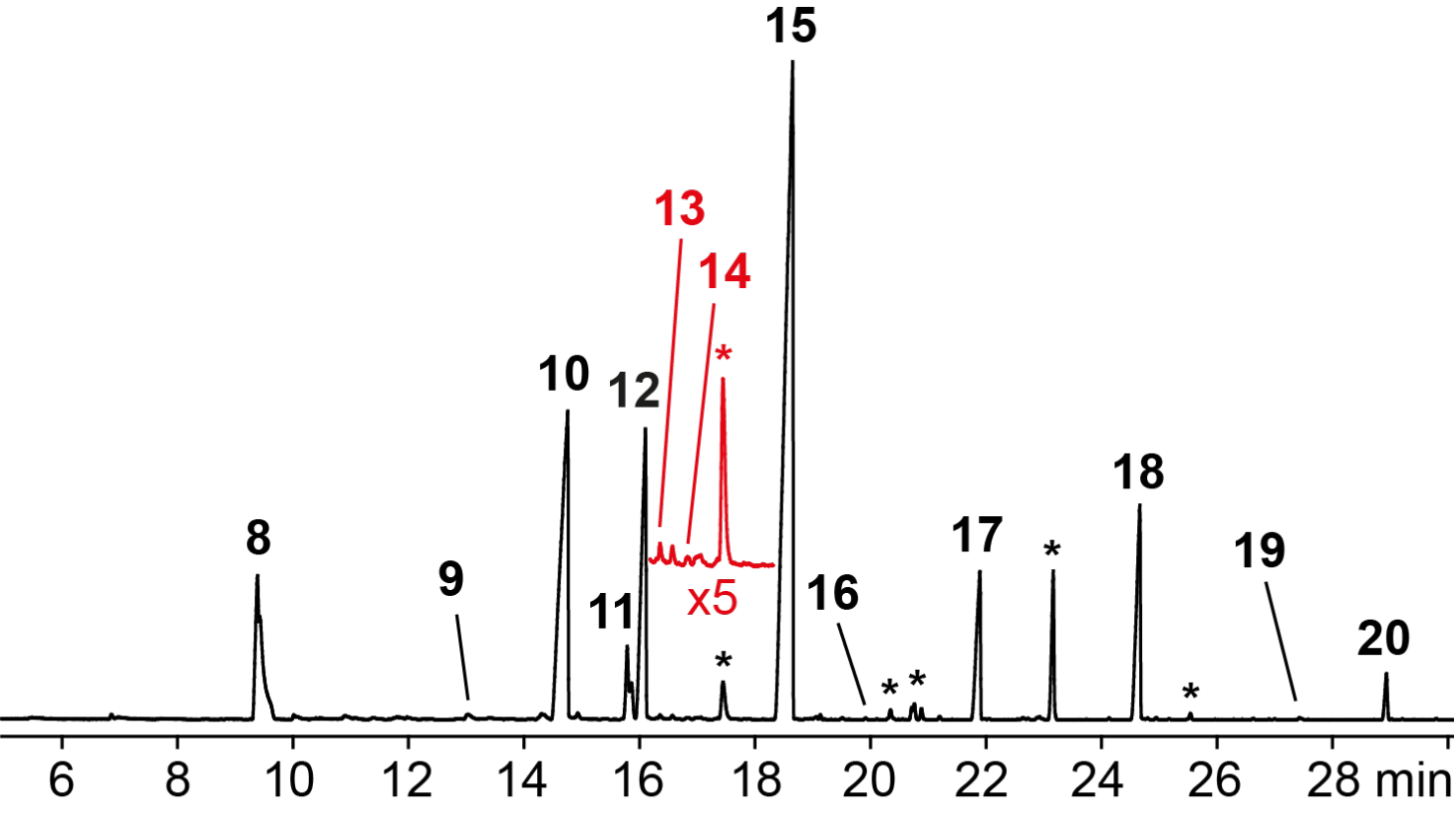
Total-ion chromatogram of the bouquet from *Hypoxylon invadens* MUCL 54175 obtained by the CLSA headspace technique. Numbers at peaks correspond to the compounds in Table 1 and Figure 3, asterisks indicate unknown compounds. The red line shows a 500% superelevation of the chromatogram between 16.2 min and 18.3 min.

**Figure 3:**
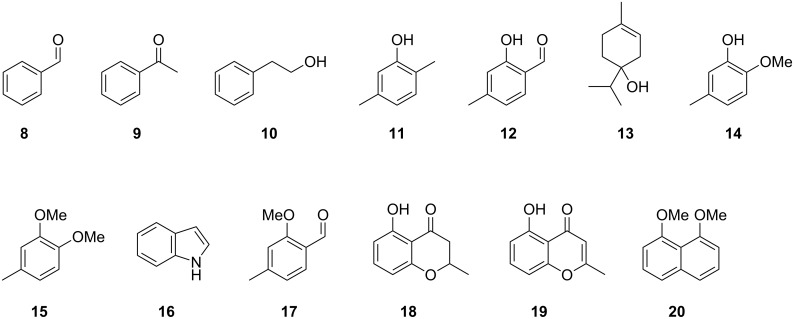
Identified volatile organic compounds from *Hypoxylon invadens* MUCL 54175.

The volatiles released by agar plate cultures of *Hypoxylon invadens* MUCL 54175 were collected through the CLSA headspace method and the obtained headspace extracts were analysed by GC–MS. The gas chromatogram of a representative extract is shown in Figure 2. Several volatiles could immediately be identified based on their mass spectra and retention indices (Table 1 and Figure 3), including the major compounds benzaldehyde (**8**) and 2-phenylethanol (**10**), and the trace components acetophenone (**9**), terpinen-4-ol (**13**), and indole (**16**). For several other compounds in the headspace extract close hits for highly substituted aromatic compounds were found in our mass spectral libraries, but the mass spectra and retention indices for some of these compounds also showed some differences. Furthermore, the structures of regioisomers with different substitution patterns at the aromatic ring could not be excluded, because the mass spectra and retention indices were not available for all these compounds.

**Table 1:** Volatiles identified in the bouquet of *Hypoxylon invadens* MUCL 54175.

Compound	*I*	*I* (Ref.)	Identification^a^	Peak area^b^

benzaldehyde (**8**)	961	951 [14]	ms, ri, std	7.9%
acetophenone (**9**)	1066	1059 [14]	ms, ri, std	0.2%
2-phenylethanol (**10**)	1111	1106 [14]	ms, ri, std	18.1%
2,5-dimethylphenol (**11**)	1152	1151 [15]	ms, ri, std	2.4%
2-hydroxy-4-methylbenzaldehyde (**12**)	1165		std	10.6%
terpinen-4-ol (**13**)	1179	1174 [14]	ms, ri	0.1%
2-methoxy-5-methylphenol (**14**)	1187		std	<0.1%
3,4-dimethoxytoluene (**15**)	1240		std	40.6%
indole (**16**)	1293	1290 [14]	ms, ri, std	<0.1%
2-methoxy-4-methylbenzaldehyde (**17**)	1364		syn	4.7%
5-hydroxy-2-methylchroman-4-one (**18**)	1472		ms	6.8%
5-hydroxy-2-methyl-4*H*-chromen-4-one (**19**)	1591		syn	0.1%
1,8-dimethoxynaphthalene (**20**)	1657		ms	1.0%

^a^Compound identification by ms: mass spectrum identical to a library spectrum, ri: retention index identical to published data, std: comparison to an authentic standard, syn: comparison to a compound synthesised in this work. ^b^Peak integral (% of sum of peak integrals). Medium components, contaminants such as plasticisers and unknown compounds are not listed.

**Figure 4:**
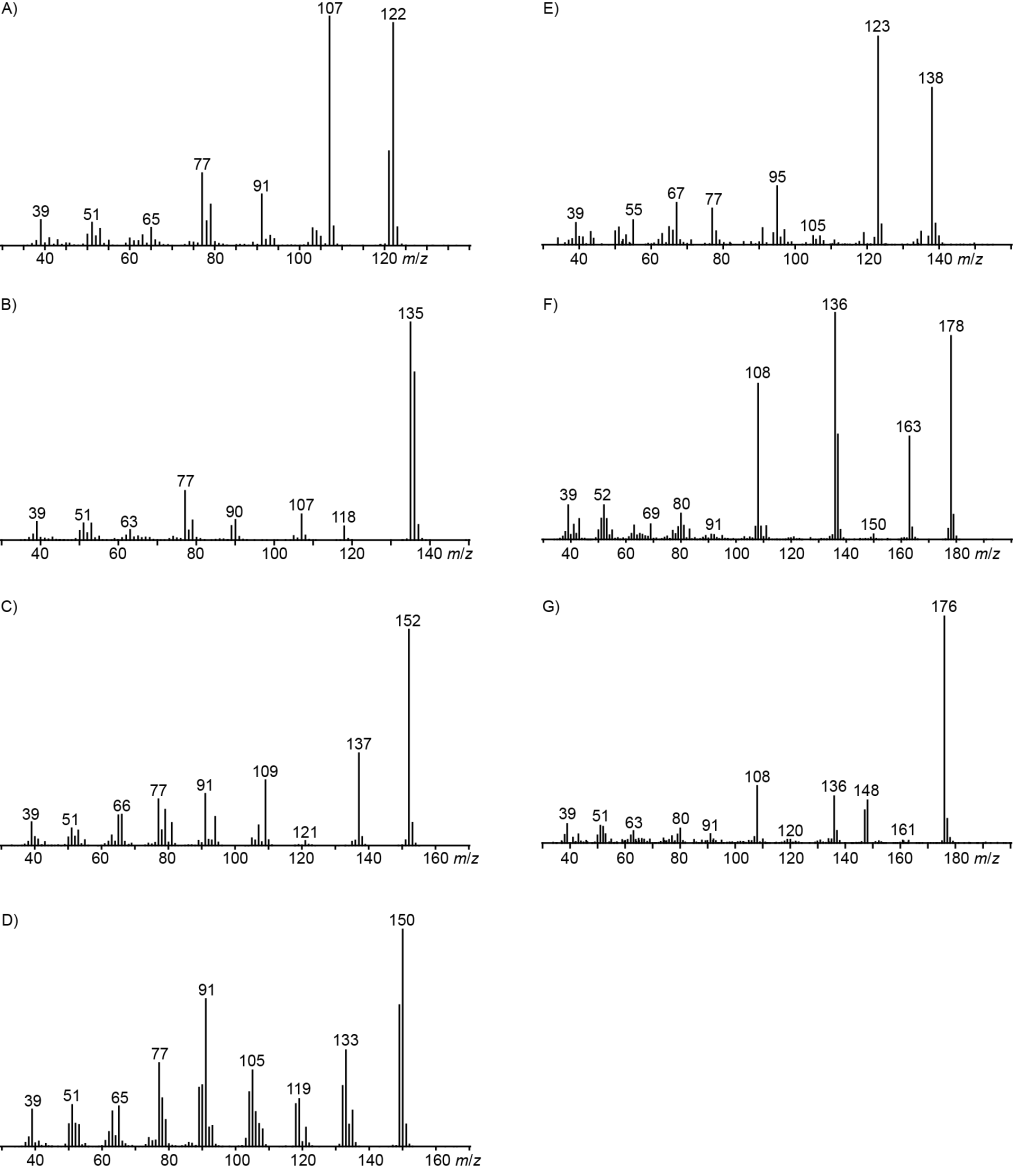
Mass spectra of volatiles from *Hypoxylon invadens* MUCL 54175. Mass spectra of A) 2,5-dimethylphenol (**11**), B) 2-hydroxy-4-methylbenzaldehyde (**12**), C) 3,4-dimethoxytoluene (**15**), D) 2-methoxy-4-methylbenzaldehyde (**17**), E) 2-methoxy-5-methylphenol (**14**), F) 5-hydroxy-2-methylchroman-4-one (**18**), and G) 5-hydroxy-2-methyl-4*H*-chromen-4-one (**19**).

The mass spectrum of one of these compounds (**11**, Figure 4A) suggested the structure of a dimethylphenol, but the mass spectra of several regioisomers in our database proved to be nearly identical. The measured retention index (*I* = 1152) matched with a published retention index for 2,5-dimethylphenol (*I* = 1151) [15], but retention indices were not available for all the possible molecules. Six constitutional isomers with different substitution patterns at the aromatic ring exist for dimethylphenol that were all commercially available. A direct comparison by GC–MS unequivocally established the identity of **11** and 2,5-dimethylphenol by identical retention index and best matching mass spectrum (Table 2).

**Table 2:** Retention indices of all regioisomers of dimethylphenol.

Structure	Compound name	*I*	MS match^a^

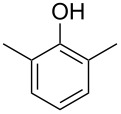	2,6-dimethylphenol	1108	862
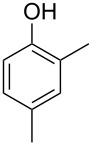	2,4-dimethylphenol	1147	879
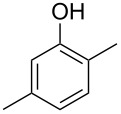	2,5-dimethylphenol (**11**)	1152	913
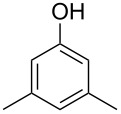	3,5-dimethylphenol	1164	903
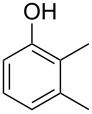	2,3-dimethylphenol	1177	901
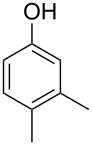	3,4-dimethylphenol	1191	892

^a^MS match of the mass spectrum of the natural product from *H. invadens* in comparison to the mass spectrum of the tabulated compound (a value of 1000 would indicate identical mass spectra, positive compound identification can usually be assumed for a value >900).

The mass spectrum of the second compound **12** (Figure 4B) was very similar to the mass spectra of several regioisomers of hydroxy-methylbenzaldehydes included in our libraries, but retention indices for the complete set of regioisomers were not available from the literature. Also in this case all ten constitutional isomers of hydroxy-methylbenzaldehyde were commercially available and a comparison of the headspace constituent from *H. invadens* to all these compounds by GC–MS allowed for the unambiguous identification of **12** as 2-hydroxy-4-methylbenzaldehyde (Table 3). The best MS match was obtained for 2-hydroxy-6-methylbenzaldehyde (MS match: 904), but the mass spectrum of 2-hydroxy-4-methylbenzaldehyde produced a match that was nearly as good (MS match: 902), and the structure of 2-hydroxy-6-methylbenzaldehyde could clearly be excluded by a different retention index.

**Table 3:** Retention indices of all regioisomers of hydroxy-methylbenzaldehyde.

Structure	Compound name	*I*	MS match^a^

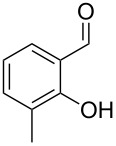	2-hydroxy-3-methylbenzaldehyde	1139	902
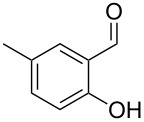	2-hydroxy-5-methylbenzaldehyde	1160	893
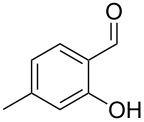	2-hydroxy-4-methylbenzaldehyde (**12**)	1165	902
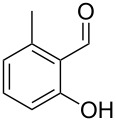	2-hydroxy-6-methylbenzaldehyde	1202	904
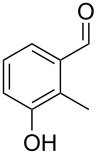	3-hydroxy-2-methylbenzaldehyde	1374	878
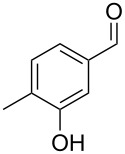	3-hydroxy-4-methylbenzaldehyde	1396	874
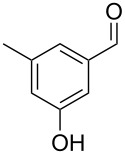	3-hydroxy-5-methylbenzaldehyde	1399	857
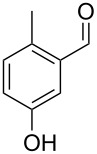	3-hydroxy-6-methylbenzaldehyde	1408	785
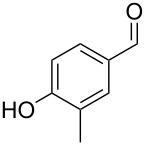	4-hydroxy-3-methylbenzaldehyde	1427	857
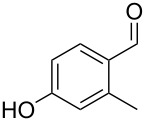	4-hydroxy-2-methylbenzaldehyde	1452	857

^a^MS match of the mass spectrum of the natural product from *H. invadens* in comparison to the mass spectrum of the tabulated compound (a value of 1000 would indicate identical mass spectra, positive compound identification can usually be assumed for a value >900).

The main compound **15** released by *H. invadens* exhibited a mass spectrum that pointed to the structure of a dimethoxytoluene (Figure 4C), but again the mass spectra of various different regioisomers in our mass spectral libraries were too similar to distinguish between the possibilities and retention indices were not available for all cases. All six constitutional isomers were obtained from commercial suppliers and compared to the natural product, establishing the identity of **15** and 3,4-dimethoxytoluene, for which an identical retention index and the best MS match was determined (Table 4).

**Table 4:** Retention indices of all regioisomers of dimethoxytoluene.

Structure	Compound name	*I*	MS match^a^

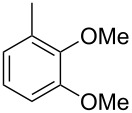	2,3-dimethoxytoluene	1176	905
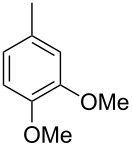	3,4-dimethoxytoluene (**15**)	1240	937
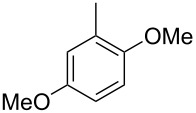	2,5-dimethoxytoluene	1252	772
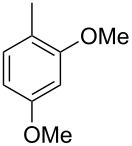	2,4-dimethoxytoluene	1257	742
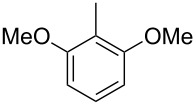	2,6-dimethoxytoluene	1259	779
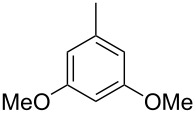	3,5-dimethoxytoluene	1271	716

^a^MS match of the mass spectrum of the natural product from *H. invadens* in comparison to the mass spectrum of the tabulated compound (a value of 1000 would indicate identical mass spectra, positive compound identification can usually be assumed for a value >900).

Similarly, the mass spectrum of compound **17** hinted to the structure of a methoxy-methylbenzaldehyde isomer (Figure 4D), for which like for the hydroxy-methylbenzaldehydes ten different constitutional isomers are possible. For an unambiguous structural assignment all ten commercially obtained hydroxy-methylbenzaldehydes were converted into the corresponding methoxy derivatives by methylation with potassium carbonate and methyl iodide. The GC–MS analysis of all the obtained methylation products unequivocally established the identity of **17** and 2-methoxy-4-methylbenzaldehyde by the same retention index and best matching mass spectrum (Table 5).

**Table 5:** Retention indices of all regioisomers of methoxy-methylbenzaldehyde.

Structure	Compound name	*I*	MS match^a^

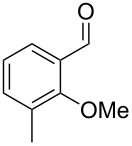	2-methoxy-3-methylbenzaldehyde	1265	894
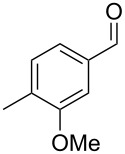	3-methoxy-4-methylbenzaldehyde	1307	697
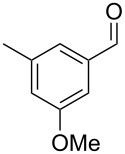	3-methoxy-5-methylbenzaldehyde	1313	670
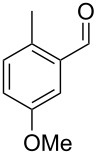	3-methoxy-6-methylbenzaldehyde	1323	650
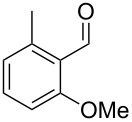	2-methoxy-6-methylbenzaldehyde	1335	889
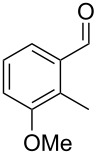	3-methoxy-2-methylbenzaldehyde	1335	741
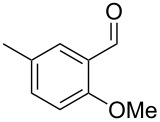	2-methoxy-5-methylbenzaldehyde	1352	884
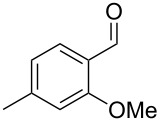	2-methoxy-4-methylbenzaldehyde (**17**)	1364	934
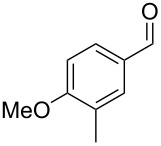	4-methoxy-3-methylbenzaldehyde	1366	660
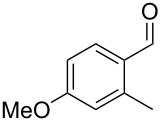	4-methoxy-2-methylbenzaldehyde	1368	628

^a^MS match of the mass spectrum of the natural product from *H. invadens* in comparison to the mass spectrum of the tabulated compound (a value of 1000 would indicate identical mass spectra, positive compound identification can usually be assumed for a value >900).

**Scheme 1:**
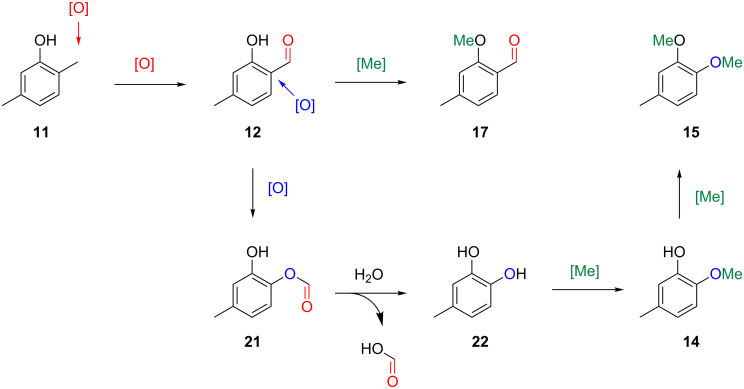
Proposed common biosynthetic pathway to volatile aromatic compounds from *Hypoxylon invadens*.

Notably, a common biosynthesis for all the identified aromatic compounds can be assumed that further strengthens their structure elucidations (Scheme 1). Starting from **11**, an oxidation step at the 2-methyl group (red) could lead via the benzyl alcohol derivative to the corresponding aldehyde **12** that upon O-methylation (green), likely with *S*-adenosylmethionine, would result in **17**. The alternative oxidation of **12** by a Baeyer–Villiger monooxygenase could result in the insertion of an oxygen (blue) to yield the formate ester **21**, followed by ester hydrolysis to 4-methylcatechol (**22**). Two sequential O-methylations (green) could give rise to **15** via the hypothetical intermediate 2-methoxy-5-methylphenol (**14**). These thoughts prompted us to search for missing biosynthetic links in the headspace extracts of *Hypoxylon invadens*. Indeed, a trace compound with a mass spectrum that could fit to the structure of **14** was observed (Figure 4E). This compound exhibited the same retention index and mass spectrum as a commercially available standard of 2-methoxy-5-methylphenol. Unfortunately, not all the constitutional isomers with different ring substitution patterns were available from standard suppliers of fine chemicals and thus the structure of another regioisomer cannot fully be excluded for **14**, but the closure of the biosynthetic gap by 2-methoxy-5-methylphenol intriguingly favours this structural assigment for **14**. The alternative structure of 2-methoxy-4-methylphenol that would be an intermediate if the methylations would proceed by a reverse order of steps was excluded for the detected compound, because the mass spectrum of 2-methoxy-4-methylphenol was included in our libraries and showed significant differences.

The compound 5-hydroxy-2-methylchroman-4-one (**18**), one of the major constituents in the *H. invadens* headspace extracts, has been reported before from various ascomycetes, especially from the genus *Daldinia* and other endophytic fungi [16–25]. It did, however, not constitute a major metabolite during the screening of numerous other species of *Hypoxylon* and the related *Annulohypoxylon* using HPLC/DAD–MS detection of organic extracts from standardised submerged cultures [26].

**Scheme 2:**
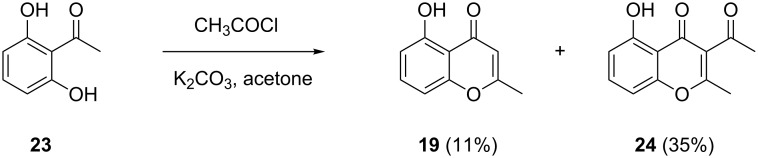
Synthesis of 5-hydroxy-2-methyl-4*H*-chromen-4-one (**19**).

Another of the trace components (**19**) displayed a molecular ion that was reduced by 2 Da in comparison to the molecular ion of **18** (compare Figure 4F and 4G), and the mass spectrum of this compound was highly similar to a mass spectrum of 5-hydroxy-2-methyl-4*H*-chromen-4-one in our mass spectral library. A synthetic standard of this compound was obtained via a known procedure from 2,6-dihydroxyacetophenone (**23**) and acetyl chloride under basic conditions [27], with formation of 3-acetyl-5-hydroxy-2-methyl-4*H*-chromen-4-one (**24**) as the main product (Scheme 2). The synthetic 5-hydroxy-2-methyl-4*H*-chromen-4-one proved to be identical to natural **19** in terms of its gas chromatographic behaviour and mass spectrum. Compound **19** has also been reported from other fungi before [18,21,26,28–29]. Finally, 1,8-dimethoxynaphthalene (**20**) was tentatively identified from its mass spectrum. This compound is known from another endophytic *Hypoxylon* sp. that was isolated from the Formosan plant *Litsea akoensis* var. *chitouchiaoensis* [30], and was previously reported from the fungi *Nodulisporium* sp. [18,28], *Sporothrix* sp. [31], and *Leptographium wageneri* [32]. The compound comprises the bis-methylation product of 1,8-dihydroxynaphthalene, an important precursor of melanin-type pigments in fungi [33], while **20** has been reported to inhibit melanin biosynthesis in fungi [34].

## Conclusion

The genus Hypoxylon has already been examined extensively for secondary metabolite production, and some of its species like *H. pulicicidum* and *H. rickii* are extremely creative with respect to the production of non-volatile compounds [35–37]. The current study is the first to embark on the volatiles from a fungus that was assigned with certainty to this genus. A few other recent papers were dealing with volatile secondary metabolites from endophytic isolates, but these were only tentatively assigned to the genus *Hypoxylon*, on the basis of generation of internal transcribed spacer (ITS) DNA sequences and homology comparisons [38–40]. In fact, the ITS is not regarded species- or even genus-specific in Xylariales and the phylogenies based on this DNA locus have led to rather ambiguous results. It should be interesting to find out in the future which other species of *Hypoxylon* produce the volatiles that were detected here in the apparently rare and aberrant species *H. invadens*. This fungus represents one of a few cases of mycophilic Xylariales that parasitise related species [10], whereas the vast majority of the species in this order are known to be saprotrophs with an endophytic state in their life cycle. A respective study embarking on the chemotaxonomic significance of the detected volatiles, paired with the evaluation of the biological activities of these metabolites, appears very promising to complement our knowledge on the functional diversity of the secondary metabolome of the Hypoxylaceae and other Xylariales.

## Experimental

### Strain and culture conditions

*Hypoxylon invadens* MUCL 54175 was isolated from the ascospores of the holotype specimen using the methodology described by Kuhnert et al. [41] and grown under identical conditions on YMG medium as described by Pažoutová et al. [13].

### Analysis of volatiles

The volatiles released by *H. invadens* agar plate cultures were collected using a closed-loop stripping apparatus (CLSA) [6] for 16 to 24 hours at room temperature and under circadian light-dark rhythm. The CLSA charcoal filters were extracted with HPLC grade CH_2_Cl_2_ (50 μL) and the obtained extracts were immediately analysed by GC–MS.

### GC–MS

GC–MS analyses were performed with an Agilent 7890A GC and an Agilent 5975C inert mass detector (Hewlett-Packard Company, Wilmington, USA). The GC was equipped with a non-polar HP5-MS fused silica capillary column (30 m, 0.25 mm i. d., 0.25 μm film, Agilent). Conditions were inlet pressure: 77.1 kPa, He 23.3 mL min^−1^; injection volume: 1.5 μL; injector operation mode: splitless (60 s valve time); carrier gas: He at 1.2 mL min^−1^; GC program: 5 min at 50 °C, then increasing with 5 °C min^−1^ to 320 °C; transfer line 300 °C; electron energy 70 eV. Retention indices (*I*) were determined from a homologous series of *n*-alkanes (C_8_–C_38_).

### Preparation of methoxy-methylbenzaldehydes

To a solution of the hydroxy-methylbenzaldehyde (34 mg, 0.25 mmol) in anhydrous DMF (6.0 mL), K_2_CO_3_ (35 mg, 0.25 mmol) was added and the mixture was stirred at room temperature for 30 minutes. Then, methyl iodide (30 μL, 68 mg, 0.5 mmol) was added and the reaction was stirred at room temperature overnight. The reaction was quenched by the addition of distilled water, and the aqueous phase was extracted three times with ethyl acetate. The combined organic phases were dried with MgSO_4_ and concentrated in vacuo. The residue was purified by column chromatography on silica gel.

**2-Methoxy-3-methylbenzaldehyde.** Yield: 26 mg (0.17 mmol, 69%), colourless oil. TLC (cyclohexane/ethyl acetate 10:1): *R_f_* = 0.18; ^1^H NMR (400 MHz, CDCl_3_, TMS) δ 10.38 (d, ^4^*J*_H,H_ = 0.8 Hz, 1H, CHO), 7.68 (d, ^3^*J*_H,H_ = 7.7 Hz, 1H, CH), 7.44 (d, ^3^*J*_H,H_ = 7.4 Hz, 1H, CH), 7.13 (dd, ^3^*J*_H,H_ = 7.7, 7.4 Hz, 1H, CH), 3.88 (s, 3H, OCH_3_), 2.34 (s, 3H, CH_3_) ppm; ^13^C NMR (100 MHz, CDCl_3_, TMS) δ 190.4 (CHO), 161.8 (C_q_), 137.7 (CH), 132.4 (C_q_), 129.3 (C_q_), 126.6 (CH), 124.5 (CH), 63.2 (OCH_3_), 15.6 (CH_3_) ppm; MS (EI, 70 eV) *m*/*z* (%): 150 (100) [M]^+^, 149 (31), 135 (29), 134 (10), 133 (49), 132 (52), 121 (10), 119 (12), 118 (10), 105 (26), 104 (18), 91 (47), 90 (26), 89 (18), 78 (13), 77 (26).

**2-Methoxy-4-methylbenzaldehyde (17).** Yield: 35 mg (0.23 mmol, 91%), colourless solid. TLC (cyclohexane/ethyl acetate 10:1): *R_f_* = 0.18; ^1^H NMR (400 MHz, CDCl_3_, TMS) δ 10.38 (s, 1H, CHO), 7.71 (d, ^3^*J*_H,H_ = 7.8 Hz, 1H, CH), 6.82 (d, ^3^*J*_H,H_ = 7.8 Hz, 1H, CH), 6.77 (s, 1H, CH), 3.90 (s, 3H, OCH_3_), 2.40 (s, 3H, CH_3_) ppm; ^13^C NMR (100 MHz, CDCl_3_, TMS) δ 189.6 (CHO), 162.0 (C_q_), 147.5 (C_q_), 128.7 (CH), 122.8 (C_q_), 121.8 (CH), 112.3 (CH), 55.7 (OCH_3_), 22.4 (CH_3_) ppm; MS (EI, 70 eV) *m*/*z* (%): 150 (100) [M]^+^, 149 (60), 135 (11), 133 (39), 132 (26), 118 (28), 105 (21), 104 (15), 91 (37), 90 (15), 89 (13), 77 (15).

**2-Methoxy-5-methylbenzaldehyde.** Yield: 33 mg (0.22 mmol, 89%), colourless oil. TLC (cyclohexane/ethyl acetate 10:1): *R_f_* = 0.15; ^1^H NMR (400 MHz, CDCl_3_, TMS) δ 10.43 (d, ^4^*J*_H,H_ = 0.7 Hz, 1H, CHO), 7.61 (d, ^4^*J*_H,H_ = 2.4 Hz, 1H, CH), 7.34 (dd, ^3^*J*_H,H_ = 8.5 Hz, ^4^*J*_H,H_ = 2.4 Hz, 1H, CH), 6.88 (d, ^3^*J*_H,H_ = 8.5 Hz, 1H, CH), 3.89 (s, 3H, OCH_3_), 2.30 (s, 3H, CH_3_) ppm; ^13^C NMR (100 MHz, CDCl_3_, TMS) δ 190.1 (CHO), 160.1 (C_q_), 136.7 (CH), 130.1 (C_q_), 128.7 (CH), 124.6 (C_q_), 111.7 (CH), 55.8 (OCH_3_), 20.3 (CH_3_) ppm; MS (EI, 70 eV) *m*/*z* (%): 150 (100) [M]^+^, 149 (38), 135 (14), 133 (26), 132 (20), 121 (10), 118 (10), 105 (18), 104 (17), 91 (33), 90 (13), 89 (13), 77 (19).

**2-Methoxy-6-methylbenzaldehyde.** Yield: 27 mg (0.18 mmol, 72%), colourless solid. TLC (cyclohexane/ethyl acetate 10:1): *R_f_* = 0.29; ^1^H NMR (400 MHz, CDCl_3_, TMS): δ 10.64 (s, CHO), 7.37 (dd, ^3^*J*_H,H_ = 8.4, 7.6 Hz, 1H, CH), 6.83 (d, ^3^*J*_H,H_ = 8.4 Hz, 1H, CH), 6.79 (dq, ^3^*J*_H,H_ = 7.6 Hz, ^4^*J*_H,H_ = 0.8 Hz, 1H, CH), 3.89 (s, 3H, OCH_3_), 2.56 (s, 3H, CH_3_) ppm; ^13^C NMR (100 MHz, CDCl_3_, TMS) δ 192.4 (CHO), 163.3 (C_q_), 142.1 (C_q_), 134.6 (CH), 124.2 (CH), 123.5 (C_q_), 109.2 (CH), 55.9 (OCH_3_), 21.6 (CH_3_) ppm; MS (EI, 70 eV) *m*/*z* (%): 150 (100) [M]^+^, 151 (10), 149 (47), 135 (16), 134 (6), 133 (27), 132 (13), 119 (5), 118 (15), 105 (13), 104 (7), 91 (30), 90 (19), 89 (12), 79 (6), 78 (9), 77 (14).

**3-Methoxy-2-methylbenzaldehyde.** Yield: 27 mg (0.18 mmol, 72%), pale yellow oil. TLC (cyclohexane/ethyl acetate 10:1): *R_f_* = 0.24; ^1^H NMR (400 MHz, CDCl_3_, TMS) δ 10.64 (d, ^4^*J*_H,H_ = 0.6 Hz, 1H, CHO), 7.37 (dd, ^3^*J*_H,H_ = 8.4, 7.6 Hz, 1H, CH), 6.83 (d, ^3^*J*_H,H_ = 8.4 Hz, 1H, CH), 6.80 (d, ^3^*J*_H,H_ = 7.6 Hz, 1H, CH), 3.89 (s, 3H, OCH_3_), 2.56 (s, 3H, CH_3_) ppm; ^13^C NMR (100 MHz, CDCl_3_, TMS) δ 192.4 (CHO), 163.4 (C), 142.1 (C), 134.6 (CH), 124.2 (CH), 123.5 (C), 109.2 (CH), 55.9 (OCH_3_), 21.6 (CH_3_) ppm; MS (EI, 70 eV) *m*/*z* (%): 150 (100) [M]^+^, 151 (9), 149 (40), 135 (5), 121 (15), 120 (12), 119 (10), 107 (8), 105 (8), 91 (39), 79 (7), 78 (7), 77 (20), 51 (5).

**3-Methoxy-4-methylbenzaldehyde.** Yield: 35 mg (0.23 mmol, 91%), colourless solid. TLC (cyclohexane/ethyl acetate 10:1): *R_f_* = 0.18; ^1^H NMR (400 MHz, CDCl_3_, TMS) δ 9.92 (s, 1H, CHO), 7.36 (dd, ^3^*J*_H,H_ = 7.5 Hz, ^4^*J*_H,H_ = 1.5 Hz, 1H, CH), 7.33 (d, ^4^*J*_H,H_ = 1.4 Hz, 1H, CH), 7.29 (d, ^3^*J*_H,H_ = 7.5 Hz, 1H, CH), 3.89 (s, 3H, OCH_3_), 2.29 (s, 3H, CH_3_) ppm; ^13^C NMR (100 MHz, CDCl_3_, TMS) δ 192.1 (CHO), 158.5 (C_q_), 136.0 (C_q_), 135.0 (C_q_), 131.0 (CH), 124.6 (CH), 108.0 (CH), 55.6 (OCH_3_), 17.0 (CH_3_) ppm; MS (EI, 70 eV) *m*/*z* (%): 150 (100) [M]^+^, 151 (9), 149 (89), 135 (4), 122 (4), 121 (22), 106 (4), 91 (25), 89 (3), 79 (3), 78 (5), 77 (13), 65 (4), 51 (3), 44 (3).

**3-Methoxy-5-methylbenzaldehyde.** Yield: 28 mg (0.19 mmol, 75%), pale yellow oil. TLC (cyclohexane/ethyl acetate 10:1): *R_f_* = 0.23; ^1^H NMR (400 MHz, CDCl_3_, TMS) δ 9.92 (s, 1H, CHO), 7.26 (s, 1H, CH), 7.19 (br s, 1H, CH), 6.98 (m, 1H, CH), 3.84 (s, 3H, OCH_3_), 2.39 (d, ^4^*J*_H,H_ = 0.9 Hz, 3H, CH_3_) ppm; ^13^C NMR (100 MHz, CDCl_3_, TMS) δ 192.5 (CHO), 160.3 (C_q_), 140.5 (C_q_), 137.9 (C_q_), 124.5 (CH), 122.3 (CH), 109.6 (CH), 55.6 (OCH_3_), 21.3 (CH_3_) ppm; MS (EI, 70 eV) *m*/*z* (%): 150 (100) [M]^+^, 151 (9), 149 (85), 122 (5), 121 (33), 119 (3), 106 (3), 105 (2), 92 (2), 91 (16), 89 (2), 79 (3), 78 (4), 77 (10), 65 (3), 63 (2), 51 (2).

**3-Methoxy-6-methylbenzaldehyde.** Yield: 21 mg (0.14 mmol, 56%), colourless oil. TLC (cyclohexane/ethyl acetate 10:1): *R_f_* = 0.19; ^1^H NMR (400 MHz, CDCl_3_, TMS) δ 10.27 (s, 1H, CHO), 7.32 (d, ^4^*J*_H,H_ = 2.9 Hz, 1H, CH), 7.16 (d, ^3^*J*_H,H_ = 8.4 Hz, 1H, CH), 7.04 (dd, ^3^*J*_H,H_ = 8.4 Hz, ^4^*J*_H,H_ = 2.9 Hz, 1H, CH), 3.84 (s, 3H, OCH_3_), 2.60 (s, 3H, CH_3_) ppm; ^13^C NMR (100 MHz, CDCl_3_, TMS) δ 192.2 (CHO), 158.2 (C_q_), 134.8 (C_q_), 133.0 (C_q_), 132.9 (CH), 120.9 (CH), 114.2 (CH), 55.6 (OCH_3_), 18.2 (CH_3_) ppm; MS (EI, 70 eV) *m*/*z* (%): 150 (100) [M]^+^, 151 (9), 149 (45), 135 (4), 122 (9), 121 (68), 119 (2), 107 (7), 106 (2), 92 (2), 91 (13), 89 (3), 79 (4), 78 (6), 77 (14), 65 (3), 63 (2), 51 (3).

**4-Methoxy-2-methylbenzaldehyde.** Yield: 32 mg (0.21 mmol, 84%), pale yellow oil. TLC (cyclohexane/ethyl acetate 10:1): *R_f_* = 0.18; ^1^H NMR (400 MHz, CDCl_3_, TMS) δ 10.11 (s, 1H, CHO), 7.75 (d, ^3^*J*_H,H_ = 8.6 Hz, 1H, CH), 6.84 (dd, ^3^*J*_H,H_ = 8.5 Hz, ^4^*J*_H,H_ = 2.5 Hz, 1H, CH), 6.74 (d, ^4^*J*_H,H_ = 2.5 Hz, 1H, CH), 3.86 (s, 3H, OCH_3_), 2.65 (s, 3H, CH_3_) ppm; ^13^C NMR (100 MHz, CDCl_3_, TMS) δ 191.3 (CHO), 163.8 (C_q_), 143.4 (C_q_), 134.9 (CH), 128.1 (C_q_), 117.1 (CH), 111.6 (CH), 55.6 (OCH_3_), 20.0 (CH_3_) ppm; MS (EI, 70 eV) *m*/*z* (%): 150 (65) [M]^+^, 151 (6), 149 (100), 122 (3), 121 (17), 106 (2), 91 (12), 89 (2), 78 (4), 77 (9), 63 (2), 51 (3).

**4-Methoxy-3-methylbenzaldehyde.** Yield: 32 mg (0.21 mmol, 84%), colourless solid. TLC (cyclohexane/ethyl acetate 10:1): *R_f_* = 0.15; ^1^H NMR (400 MHz, CDCl_3_, TMS) δ 9.84 (s, 1H, CHO), 7.70 (dd, ^3^*J*_H,H_ = 8.4 Hz, ^4^*J*_H,H_ = 2.2 Hz, 1H, CH), 7.67 (d, ^4^*J*_H,H_ = 1.8 Hz, 1H, CH), 6.91 (d, ^3^*J*_H,H_ = 8.4 Hz, 1H, CH), 3.90 (s, 3H, OCH_3_), 2.25 (s, 3H, CH_3_) ppm; ^13^C NMR (100 MHz, CDCl_3_, TMS) δ 191.3 (CHO), 163.0 (C_q_), 131.6 (CH), 130.8 (CH), 129.6 (C_q_), 127.8 (C_q_), 109.8 (CH), 55.8 (OCH_3_), 16.3 (CH_3_) ppm; MS (EI, 70 eV) *m*/*z* (%): 150 (69) [M]^+^, 151 (6), 149 (100), 121 (7), 106 (3), 91 (17), 89 (2), 78 (3), 77 (8), 65 (2).

### Synthesis of 5-hydroxy-2-methyl-4*H*-chromen-4-one (**19**)

According to Okombi et al. [27], potassium carbonate (1.38 g, 10.0 mmol) was added to a solution of 2,6-dihydroxyacetophenone (**23**, 304 mg, 2.0 mmol) in acetone (10 mL), and the mixture was stirred at room temperature for 15 minutes. Then, acetyl chloride (157 mg, 2.0 mmol) was added and stirring was continued under reflux for 24 hours. The reaction was cooled to room temperature and hydrolysed by the addition of distilled water. The aqueous layer was extracted three times with diethyl ether. The collected organic phases were dried over MgSO_4_ and concentrated in vacuo. The residue was purified by column chromatography on silica gel to afford the title compound **19** (37 mg, 0.21 mmol, 11%) and 3-acetyl-5-hydroxy-2-methyl-4*H*-chromen-4-one (**24**) as main product (154 mg, 0.71 mmol, 35%). Both compounds were obtained as pale yellow solid.

**5-Hydroxy-2-methyl-4*H*-chromen-4-one (19).** TLC (hexane/ethyl acetate/toluene, 3:1:1): *R_f_* = 0.36; GC (HP-5): *I* = 1591; ^1^H NMR (400 MHz, CDCl_3_, TMS) δ 12.54 (s, 1H, OH), 7.48 (dd, ^3^*J*_H,H_ = 8.5, 8.3 Hz, 1H, CH), 6.84 (dd, ^3^*J*_H,H_ = 8.5 Hz, ^4^*J*_H,H_ = 0.8 Hz, 1H, CH), 6.76 (dd, ^3^*J*_H,H_ = 8.3 Hz, ^4^*J*_H,H_ = 0.8 Hz, 1H, CH), 6.09 (s, 1H, CH), 2.38 (s, 3H, CH_3_) ppm; ^13^C NMR (100 MHz, CDCl_3_) δ 183.6 (C_q_), 167.7 (C_q_), 160.9 (C_q_), 156.9 (C_q_), 135.2 (CH), 111.3 (CH), 110.6 (C_q_), 109.3 (CH), 106.9 (CH), 20.8 (CH_3_) ppm; IR (ATR) *v*: 3075 (w), 2970 (w), 2930 (w), 2850 (w), 2781 (w), 1664 (s), 1622 (s), 1596 (s), 1465 (m), 1404 (s), 1377 (s), 1305 (s), 1250 (s), 1228 (s), 1183 (m), 1158 (m), 1110 (m), 1061 (m), 1002 (m), 953 (s), 867 (m), 838 (m), 799 (s), 745 (s), 741 (m), 681 (m), 617 (m), 587 (s) cm^−1^; UV–vis (CH_2_Cl_2_): λ_max_ (lg ε) = 325 (4.64), 253 (5.08), 230 (5.30) nm; MS (EI, 70 eV) *m*/*z* (%): 176 (100) [M]^+^, 148 (32), 147 (26), 136 (22), 108 (37), 91 (10), 39 (12).

**3-Acetyl-5-hydroxy-2-methyl-4*H*-chromen-4-one (24).** TLC (hexane/ethyl acetate/toluene, 3:1:1): *R_f_* = 0.33; GC (HP-5): *I* = 1800; ^1^H NMR (400 MHz, CDCl_3_, TMS) δ 12.42 (s, 1H, OH), 7.52 (dd, ^3^*J*_H,H_ = 8.4, 8.3 Hz, 1H, CH), 6.86 (dd, ^3^*J*_H,H_ = 8.4 Hz, ^4^*J*_H,H_ = 0.9 Hz, 1H, CH), 6.80 (dd, ^3^*J*_H,H_ = 8.3 Hz, ^4^*J*_H,H_ = 0.9 Hz, 1H, CH), 2.62 (s, 3H, CH_3_), 2.52 (s, 3H, CH_3_) ppm; ^13^C NMR (100 MHz, CDCl_3_) δ 199.3 (C_q_), 181.5 (C_q_), 170.4 (C_q_), 161.1 (C_q_), 155.7 (C_q_), 135.9 (CH), 122.3 (C_q_), 112.2 (CH), 110.5 (C_q_), 106.9 (CH), 32.4 (CH_3_), 20.2 (CH_3_) ppm; IR (ATR) *v*: 3073 (w), 2965 (w), 2925 (w), 2851 (w), 1692 (s), 1642 (s), 1601 (s), 1503 (m), 1469 (s), 1409 (s), 1376 (m), 1348 (m), 1296 (s), 1213 (s), 1164 (m), 1129 (m), 1078 (m), 1057 (m), 1036 (m), 994 (m), 953 (m), 881 (m), 861 (m), 812 (s), 756 (s), 707 (s), 650 (m), 634 (m), 593 (m), 531 (m) cm^−1^; UV–vis (CH_2_Cl_2_) λ_max_ (lg ε): 328 (4.65), 241 (5.17), 228 (5.19) nm; MS (EI, 70 eV) *m*/*z* (%): 218 (81) [M]^+^, 204 (13), 203 (100), 137 (53), 136 (17), 108 (21), 67 (26), 43 (23), 39 (13).
